# Genomic Analysis of *Microbulbifer* sp. Strain A4B-17 and the Characterization of Its Metabolic Pathways for 4-Hydroxybenzoic Acid Synthesis

**DOI:** 10.3389/fmicb.2018.03115

**Published:** 2018-12-18

**Authors:** Jun Tian, Li Zhu, Wenjun Wang, Liping Zhang, Zhi Li, Qingyu Zhao, Ke Xing, Zhaozhong Feng, Xue Peng

**Affiliations:** School of Life Sciences, Jiangsu Normal University, Xuzhou, China

**Keywords:** 4-hydroxybenzoic acid, *Microbulbifer*, biosynthesis, parabens, shikimate

## Abstract

The marine bacterium *Microbulbifer* sp. A4B-17 produces secondary metabolites such as 4-hydroxybenzoic acid (4HBA) and esters of 4HBA (parabens). 4HBA is a useful material in the synthesis of the liquid crystal. Parabens are man-made compounds that have been extensively used since the 1920s in the cosmetic, pharmaceutical, and food industries for their effective antimicrobial activity. In this study, we completed the sequencing and annotation of the A4B-17 strain genome and found all genes for glucose utilization and 4HBA biosynthesis. Strain A4B-17 uses the Embden-Meyerhof-Parnas (EMP), hexose monophosphate (HMP), and Entner-Doudoroff (ED) pathways to utilize glucose. Other sugars such as fructose, sucrose, xylose, arabinose, galactose, mannitol, and glycerol supported cell growth and 4HBA synthesis. Reverse transcriptional analysis confirmed that the key genes involved in the glucose metabolism were functional. Paraben concentrations were proportionally increased by adding alcohols to the culture medium, indicating that strain A4B-17 synthesizes the 4HBA and the alcohols separately and an esterification reaction between them is responsible for the paraben synthesis. A gene that codes for a carboxylesterase was proposed to catalyze this reaction. The temperature and NaCl concentration for optimal growth were determined to be 35°C and 22.8 g/L.

## Introduction

4-Hydroxybenzoic acid (4HBA) is a simple aromatic compound that is widely used in the chemical and electrical industries as a material for the production of polymers, specifically as a monomer for the synthesis of liquid crystalline polymers (Gilkey and Caldwell, 1959). 4HBA is currently synthesized from the petroleum product benzene via cumene and phenol intermediates under high pressure and temperature with a series of chemical catalysts. The production of 4HBA from renewable resources is an effective method to solve societal problems such as environmental pollution and petroleum exhaustion. The parabens are esters of 4HBA and include methylparaben, ethylparaben, propylparaben, butylparaben, and heptylparaben. Most parabens are found naturally in plant sources. For example, methylparaben is found in *Arabidopsis thaliana*, where it acts as an antimicrobial agent (Walker et al., 2003). The parabens are widely used as preservatives by the cosmetics and pharmaceutical industries. All commercially used parabens are produced by the esterification of 4HBA with the appropriate alcohols.

Several biological pathways and production processes of 4HBA have been described in literature. 4-Hydroxycinnamoyl-CoA hydratase/lyase (HCHL) is an enoyl-CoA hydratase with an aldolase function and can convert 4-hydroxycinnamoyl-CoA to 4-hydroxybenzaldehyde (Mitra et al., 1999). Mayer et al. (2001) expressed the *Pseudomonas fluorescens* HCHL gene in tobacco cells and rerouted the plant phenylpropanoid pathway to synthesize 4HBA. A yield of 2.9% 4HBA was obtained; however, the 4HBA was modified and thus isolated as 4HBA glucoside or 4HBA glucose ester from the plant cells. Mitra et al. (1999); McQualter et al. (2005), and Rahman et al. (2009) introduced the *P. fluorescens* HCHL gene into *Datura stramonium*, sugarcane, and *Beta vulgaris*, respectively, to yield a high accumulation of the 4HBA glucose ester.

The shikimate pathway is a seven-step metabolic route used by bacteria, fungi, algae, protists, and plants for the biosynthesis of aromatic amino acids (phenylalanine, tyrosine, and tryptophan). Chorismate pyruvate lyase (CPL) catalyzes the direct conversion of chorismate, an important branch point intermediate in the shikimate pathway, to 4HBA, which is an intermediate in the biosynthesis of ubiquinone. Viitanen et al. (2004) introduced the *Escherichia coli ubiC* gene, which encodes CPL, into the tobacco chloroplast genome under the control of light. The 4HBA glucose conjugates accumulated in the oldest leaf to a peak value of 26.5% of the dry weight. A most intriguing aspect of the plant-based synthesis of 4HBA is the appeal of directly synthesizing a chemical from CO_2_. However, the glucosylation system in plant cells converted the 4HBA to glucose conjugates, making the treatment afterward difficult.

To synthesize 4HBA from the renewable carbon source glucose, Barker and Frost (2001) constructed a recombinant *E. coli* strain that overexpressed the feedback-insensitive 3-deoxy-D-arabino-heptulosonate 7-phosphate (DAHP) synthase, increased the expression of the shikimate pathway enzymes, and overexpressed the CPL gene (*E. coli ubiC*). In fed-batch fermenter conditions, the maximum concentration of 4HBA that was synthesized was 12 g/L and corresponded to a yield of 13% (mol/mol). Zhang et al. (2015) designed an *E. coli–E. coli* coculture system that achieved a production of 2.3 g/L 4HBA using a glucose and xylose mixture. One of these *E. coli* strains has the shikimate pathway for the synthesis of 3-dehydroshikimate, and the other has the ability to transport 3-dehydroshikimate and then convert it to 4HBA.

The solvent-tolerant bacterium *Pseudomonas putida* S12 was used as a platform for the production of aromatic compounds such as phenol, cinnamate, 4-coumarate, and 4HBA (Verhoef et al., 2007, 2010; Meijnen et al., 2011). The strain S12 was modified by rerouting the carbon flux from tyrosine to 4HBA, and its 4HBA hydrolase gene was inactivated to prevent further 4HBA degradation. It accumulated 4HBA at a yield of 11% on glucose in shake flask cultures. Verhoef et al. (2007) also introduced the *E. coli* xylose isomerase gene, which caused the strain to gain the ability to utilize xylose.

Krömer et al. (2013) used *Saccharomyces cerevisiae* as the platform for the overproduction of 4HBA from glucose. There are no CPL genes found in the yeast’s genome; the *E. coli ubiC* gene was introduced into *S. cerevisiae*, and the *aro7* gene responsible for the synthesis of aromatic amino acids was disrupted. These modifications led to an overproduction of 4HBA on glucose (yield 0.6%). These strains are auxotrophs that require aromatic amino acids for growth. *Corynebacterium glutamicum* was used as the platform for the overproduction of 4HBA (Kitade et al., 2018). The maximum concentration of 4-HBA produced by the engineered strain was 36.6 g/L after incubation for 24 h in minimal medium in an aerobic growth-arrested bioprocess using a jar fermentor (Kitade et al., 2018).

The genus *Microbulbifer* was established by González et al. (1997), and phylogenetic analysis of 16S rRNA gene sequences showed that this genus belongs to Gammaproteobacteria. The members of the genus *Microbulbifer* are described as Gram-negative, catalase-positive, non-motile rods. Many *Microbulbifer* strains were isolated by means of the standard dilution plating technique on Marine Broth (MB, Difco 2216) (Lee et al., 2017; Park et al., 2017). However, a few strains were isolated because they had the ability to degrade the components of marine plants, such as agar (Ohta et al., 2004; Hatada et al., 2011; Takagi et al., 2015), alginate (Wakabayashi et al., 2012), and chitin (Baba et al., 2011). In our previous study, we isolated 24 *Microbulbifer* strains from marine environments and found that they could accumulate 4HBA and its esters (parabens) in MB medium (Peng et al., 2006). A *Microbulbifer* isolate from sponge-produced parabens was also reported (Quevrain et al., 2009). It seems that the production of parabens is a common characteristic of the genus *Microbulbifer*. *Microbulbifer* sp. A4B-17 was isolated from an ascidian in the coastal waters of Palau and produces the largest amount of 4HBA (10 mg/L) and parabens compared with other strains (Peng et al., 2006). The MB medium was used in all experiments carried out in a previous study, but the source of the 4HBA and parabens was unclear. To elucidate the mechanisms of 4HBA and paraben synthesis, we used the sole carbon source in a minimum medium to grow strain A4B-17 and determined its complete genome sequence.

## Materials and Methods

### Bacterial Strains, Growth Media, and Chemicals

*Microbulbifer* sp. A4B-17 with the ability to produce 4HBA and parabens was isolated from an ascidian in the coastal waters of Palau (Peng et al., 2006). This strain was deposited in the Biological Resource Center, NITE (NBRC) with accession number NBRC101765. The nutrient medium, Difco Marine Broth 2216 (MB, 37.4 g/L), was used for general experiments. The minimum salt medium (ONR7α), supplemented with various carbohydrates, was used for testing the carbohydrate utilization of strain A4B-17. ONR7α (1 L) was composed of 22.79 g NaCl, 3.98 g Na_2_SO_4_, 0.72 g KCl, 0.27 g NH_4_Cl, 47.2 mg Na_2_HPO_4_, 83 mg NaBr, 31 mg NaHCO_3_, 27 mg H_3_BO_3_, 2.6 mg NaF, 1.118 g MgCl_2_, 0.146 g CaCl_2_⋅2H_2_O, 2.4 mg SrCl_2_⋅6H_2_O, 0.2 mg FeCl_2_⋅4H_2_O, and 1.3 g TAPSO (2-hydroxy-3-(tris(hydroxymethyl)methylamino)-1-propanesulfonic acid). The pH was adjusted to 7.6 using concentrated HCl. The chemicals used in this study, unless noted, were purchased from Sangon Biotech (Shanghai, China), and the 4HBA alkyl esters were purchased from the Tokyo Chemical Industry Co., Ltd. (Tokyo, Japan).

### Genome Sequencing and Annotation

The strain A4B-17 was cultivated in MB to the stationary phase. Its genomic DNA was extracted according to the protocols in Molecular Cloning (Sambrook and Russell, 2001). The genome was sequenced using paired-end sequencing technology (HiSeq 2000 system, Illumina, United States). The shotgun library was constructed with a 500 bp-span and a 6,000 bp-span paired-end library. Library was sequenced using MiSeq reagent kit v3 (Illumina, United States). Reads (100 bp) were assembled using SMRT Analysis 2.3.0 (Berlin et al., 2015). The gene prediction was performed using Glimmer v. 3.02 (Delcher et al., 2007), and the gene product function was annotated by BLAST using the NCBI-nr protein and Swiss-Prot databases. The classification of predicted genes and pathways were analyzed using the COGs (Tatusov et al., 2003) and KEGG (Kanehisa et al., 2016) databases.

### Growth Test With Various Carbohydrates

The strain A4B-17 was grown to the stationary phase in MB at 30°C with shaking (200 rpm), after which the cells were collected by centrifugation and washed twice with ONR7α. The resulting cells were suspended in ONR7α to give an optical density of 0.1 at 600 nm (OD_600_). Each carbohydrate was added to a final concentration of 0.2%, and the mixture was incubated at 30°C with shaking. Aliquots were withdrawn at intervals of 2 h to assess the turbidity and the production of secondary metabolites.

### Effects of Additives on Paraben Yield

The strain A4B-17 cells were prepared as stated above, in ONR7α with 0.05 OD_600_. This ONR7α medium was supplemented with 0.2% tryptone and 0.1% yeast extract. Each additive (4HBA and alcohols) was added to the medium at various concentrations (10, 20, and 30 mM). The mixture was incubated at 30°C with shaking. Aliquots were withdrawn to assess the turbidity and the production of secondary metabolites.

### Analysis of Secondary Metabolites by HPLC

A portion of the culture (500 μL) was collected at certain time intervals and mixed with an equal volume of methanol. The mixture was subjected to high-performance liquid chromatography (HPLC) after being filtered through a 0.45-μm-pore size membrane. An Alliance HPLC instrument (Waters) with an octadecylsilyl reverse-phase column (25 cm in length and 4.6 mm in diameter, Waters) was used at 40°C. A mobile phase consisting of solvent A (0.1% trifluoroacetic acid in water) and solvent B (acetonitrile) was used at a flow rate of 1 mL/min. After the injection of a sample, 90% of solvent A was run through the column for 1 min; the solvent gradient was then programmed to increase from 10 to 100% of solvent B over 15 min, and 100% solvent B was finally run for 2 min. The absorbance of the eluate was monitored at 255 nm with a photodiode array detector (Waters 2998). The respective retention times of 4HBA, methyl 4HBA, ethyl 4HBA, propyl 4HBA, butyl 4HBA, heptyl 4HBA, and nonyl 4HBA were 5.98, 8.80, 10.07, 11.34, 12.44, 15.59, and 17.36 min, respectively.

### Transcriptional Analysis

The strain A4B-17 was grown to the stationary phase in ONR7α medium supplemented with 0.2% glucose. The total RNA was isolated using a Trizol kit (Sangon Biotech), and the contaminating DNA in the sample was digested using an RNase-Free DNase Set (Qiagen). The absorbance of the RNA was measured at 260 nm with a spectrophotometer. A 300-ng amount of total RNA was used for the reverse transcriptase-polymerase chain reaction (RT-PCR), which was conducted with a Reverse Transcriptase M-MLV (Takara). The resulting cDNA was amplified using primers (Table 1).

**Table 1:** Primers used in this study.

Target gene	Forward primer	Reverse primer
GM003636	ATGCAGAAAAATATCGCCGA	GTCCAGTGCCAGGAAATCAG
GM002491	ATGAAAATTAAAATTGCCAT	CTCAATACCCAACTCACGAT
GM003580	ATGAGTCAAATTGACCAAAA	TCAATTCACTGACACCATCG
GM002069	ATGACCACACAGCAGTTCGA	CGTAACGGAAACTCCGTACT
GM001835	ATGGAAGTTGAGCTAGGAGT	CTAAATCTGCCTCAGAAATT
GM001679	ATGCAACTTCCGGGCAATGA	TTCAGACAGCATTTCTAAAG
GM001333	ATGAGCAAGATTGTCGCTGT	ATTCAACTCAGCTTCGATGC
GM000833	ATGAACAAGTTGAATCAACT	ACTCGATTCTGTAACAATAG
GM004533	GTGCCCTCTGGCGATTGGCA	CTCAGTTGCTATGTAATTGA
GM001349	ATGACTACAGACCCGGAAAT	ATATGTTGCCATAAACTGAG

### Nucleotide Sequence Accession Number

The sequence from this whole-genome shotgun project has been deposited in DDBJ/EMBL/GenBank with accession number CP029064.

## Results

### Temperature and NaCl Concentrations for Optimum Growth

**FIGURE 1:**
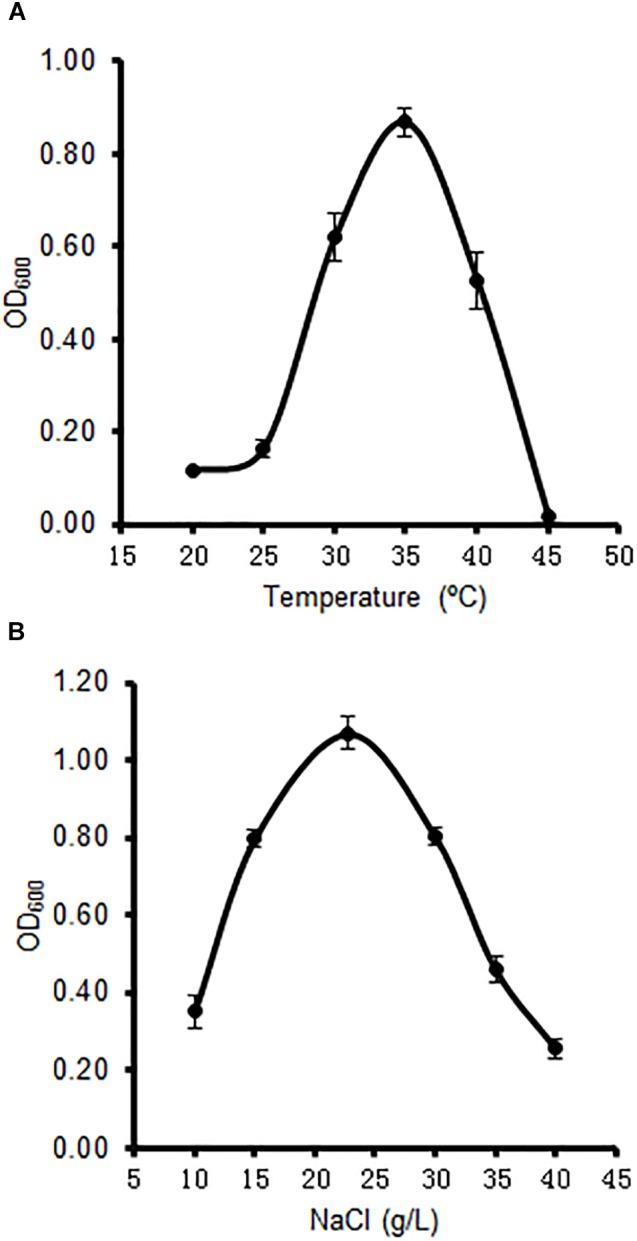
Effect of temperature **(A)** and sodium chloride **(B)** on the growth of strain A4B-17.

The strain A4B-17 was able to grow at NaCl concentrations from 15 to 30 g/L when ONR7α was used as a base medium that was supplemented with 0.1% yeast extract and 0.5% tryptone, and the optimal growth occurred at 22.8 g/L NaCl. This concentration is just the NaCl concentration in seawater, indicating that this strain is a real marine microorganism (Figure 1). The temperature range that was tested to optimize growth was from 30 to 40°C, and the optimum temperature was 35°C (Figure 1). Previous reports of the optimal temperature for members of *Microbulbifer* set the value from 30 to 37°C (Yoon et al., 2003; Ohta et al., 2004; Lee et al., 2017; Park et al., 2017). However, the optimal NaCl concentration was as low as 1–4%.

### Secondary Products Produced With Various Carbohydrates

**FIGURE 2:**
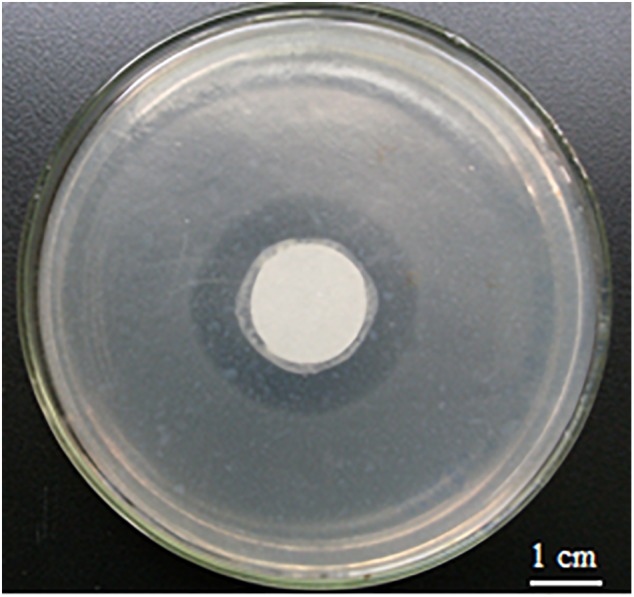
Inhibition zone of strain A4B-17 against *Bacillus subtilis*. The central circle is strain A4B-17, and the background is *Bacillus subtilis*.

When glucose was used as the sole carbon source in ONR7α, its optimal concentration for growth was 0.2%. An inhibition zone against *Bacillus subtilis* was formed by strain A4B-17 on the ONR7α agar plate that contained 0.2% glucose as the sole carbon source, indicating that strain A4B-17 could produce parabens even on the defined medium (Figure 2).

Cells of strain A4B-17 were suspended in ONR7α to OD_600_ = 0.1. To serve as the sole carbon source, glucose, fructose, glycerol, sucrose, xylose, galactose, arabinose, mannitol, alginate, and pyruvate were added to a final concentration of 0.2%, and the mixture was incubated at 30°C with shaking for 3 days. As shown in Table 2, the cell densities of all cultures reached greater than OD_600_ = 1.0, indicating that the strain A4B-17 could utilize these carbon sources for growth. 4HBA was detected in all of these cultures, and growth on sucrose produced the highest concentration of HBA, which was 18.14 μM.

**Table 2:** 4HBA produced by strain A4B-17 with various carbohydrates.

Carbon sources	OD (600 nm)	4HBA (μM)
Glucose	1.13 ± 0.03	2.64 ± 0.64
Fructose	1.14 ± 0.01	9.46 ± 0.77
Glycerol	1.19 ± 0.05	2.09 ± 0.17
Mannitol	1.11 ± 0.03	8.59 ± 0.31
Sucrose	1.20 ± 0.01	18.14 ± 0.99
Xylose	1.43 ± 0.07	8.26 ± 0.13
Alginate	1.40 ± 0.11	9.79 ± 1.04
Arabinose	1.20 ± 0.05	8.85 ± 0.02
Galactose	1.03 ± 0.05	10.22 ± 1.07
Pyruvate	1.33 ± 0.10	4.75 ± 0.67

### Effects of Additives on the Secondary Metabolite Production

**FIGURE 3:**
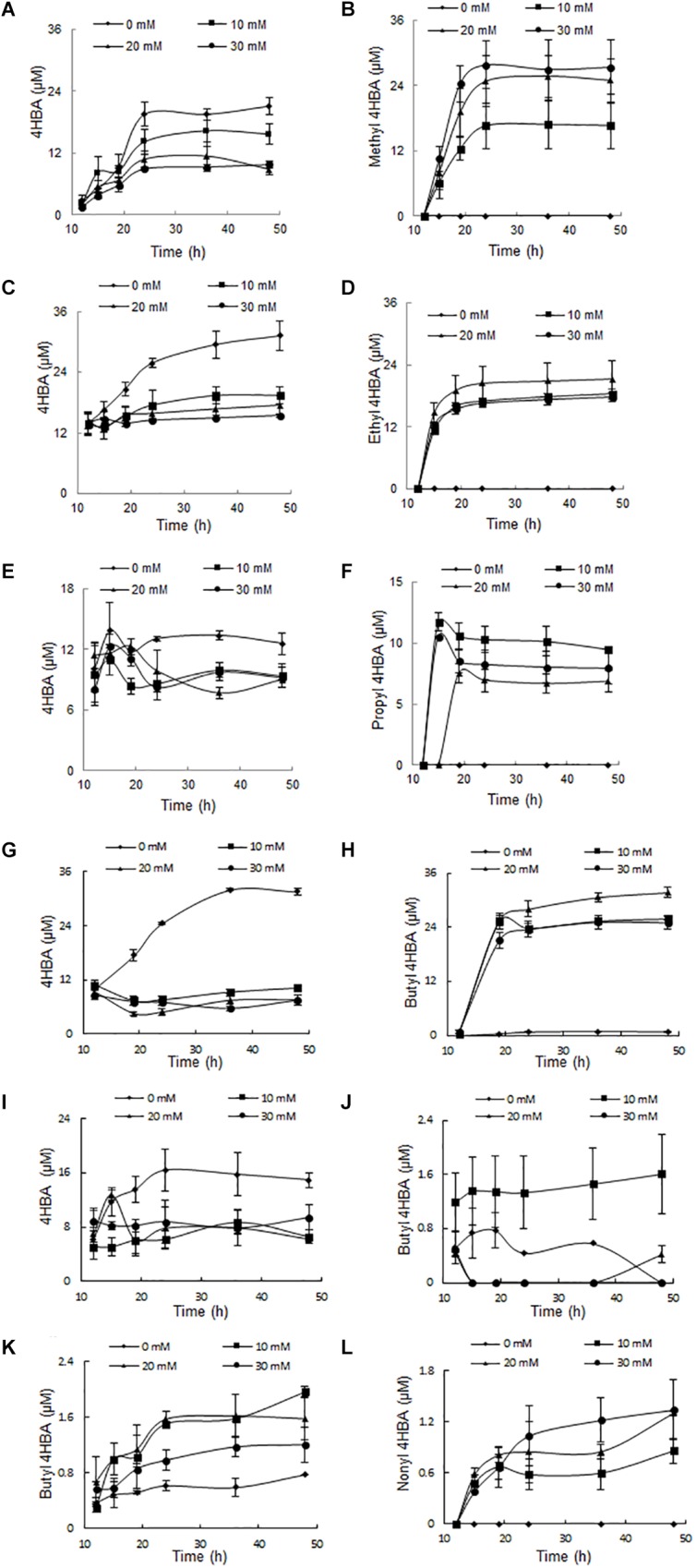
The effect of additives on metabolite accumulation. **(A)** 4HBA accumulation by adding methanol. **(B)** Methyl 4HBA accumulation by adding methanol. **(C)** 4HBA accumulation by adding ethanol. **(D)** Ethyl 4HBA accumulation by adding ethanol. **(E)** 4HBA accumulation by adding 1-propanol. **(F)** Propyl 4HBA accumulation by adding 1-propanol. **(G)** 4HBA accumulation by adding 1-butanol. **(H)** Butyl 4HBA accumulation by adding 1-butanol. **(I)** 4HBA accumulation by adding 1-heptanol. **(J)** Heptyl 4HBA accumulation by adding 1-heptanol. **(K)** Butyl 4HBA accumulation by adding 4HBA. **(L)** Nonyl 4HBA accumulation by adding 4HBA.

It is thought that 4HBA is an intermediate in the synthesis of parabens, which are formed by an esterification reaction between 4HBA and alcohols. To verify this hypothesis, each of the alcohols or 4HBA was added to the culture media, and the amount of each paraben was analyzed by HPLC after incubation for 2 days. Approximately 10 μM 4HBA was accumulated in the culture medium without additives; when methyl alcohol (10, 20, and 30 mM) was added to the culture medium, the amount of 4HBA decreased in proportion to the methyl alcohol concentration. However, the amount of methyl paraben increased in proportion to the methyl alcohol concentration (Figure 3). This phenomenon was very common to all alcohols; thus, the addition of ethyl, propyl, and butyl alcohols increased the amount of the corresponding parabens in a manner that was proportional to the alcohol’s concentration. Because heptyl and nonyl alcohols are insoluble in water, the results of the experiments with these two alcohols were not clear.

Approximately 0.2 μM butyl paraben was accumulated in the culture medium without additives; when 4HBA (10, 20, and 30 mM) was added to the culture medium, the amount of butyl paraben that was produced increased in proportion to the 4HBA concentration. Nonyl paraben was also detected when 4HBA was added (Figure 3).

The above results indicated that the strain A4B-17 synthesizes 4HBA and alcohols separately, and an esterification reaction between them is responsible for the synthesis of the parabens.

### General Features of the Strain A4B-17 Genome

The genome sequence of strain A4B-17, with a genome size of 5,035,676 bp, contains 48.73% G + C content (Supplementary Figure S1). A total of 4,604 genes were identified, representing 82.36% coding percentage, and the average gene length was 881 bp. In total, 2,206 (46.29%) genes matched a least one sequence in the COGs database when the BLASTP default parameters were used.

### Gene Expression Analysis of Strain A4B-17 Growing on Glucose

**FIGURE 4:**
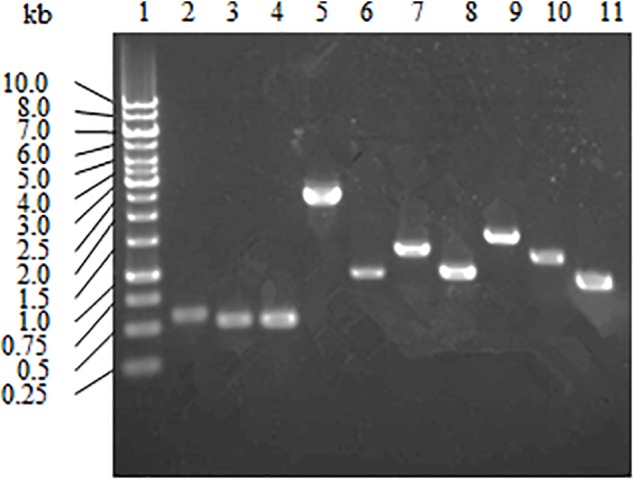
Transcriptional analysis of the genes involved in glucose utilization and 4HBA synthesis pathways. Lanes: 1, marker; 2, GM004533; 3, GM003636; 4, GM002491; 5, GM003580; 6, GM002069; 7, GM001835; 8, GM001679; 9, GM001349; 10, GM001333; and 11, GM000833.

Strain A4B-17 has three pathways, Embden-Meyerhof-Parnas (EMP), hexose monophosphate (HMP), and Entner-Doudoroff (ED), for glucose utilization. A RT-PCR analysis was applied to examine whether the genes involved in these pathways are expressed. Strain A4B-17 was grown in minimum medium with 0.2% glucose as the sole carbon and energy source, and the total RNA was extracted. The following genes were selected as the basis for primer design: GM003580 for glucose transport; GM001333, GM002491, GM003636, and GM000833 for glucose degradation; GM002069, GM001679, GM001835 for DAHP synthesis; GM004533 for 4HBA synthesis; and GM001349 for paraben synthesis. As shown in Figure 4, all selected genes were expressed, indicating that EMP, HMP, and ED pathways are functional in 4HBA biosynthesis in strain A4B-17.

## Discussion

Based on the genomic information, strain A4B-17 has three carbohydrate catabolism pathways and a complete shikimate pathway for the synthesis of 4HBA. The synthesis of parabens is proposed to occur by the esterification of 4HBA and the relevant alcohols. The key genes in each pathway were confirmed to be functional by RT-PCR analysis. Monosaccharides supported cellular growth and the syntheses of 4HBA and parabens. There are no pathways in the strain A4B-17 genome that are responsible for the degradation of aromatic compounds, such as 4HBA, vanillate, and catechol. In particular, the gene that codes for the key enzyme, the benzene ring-fissioning dioxygenase, was not found in the genome sequence of strain A4B-17. Strain A4B-17 could be cultured easily and is suitable for the construction of industrial microorganisms that biosynthesize aromatic compounds.

**FIGURE 5:**
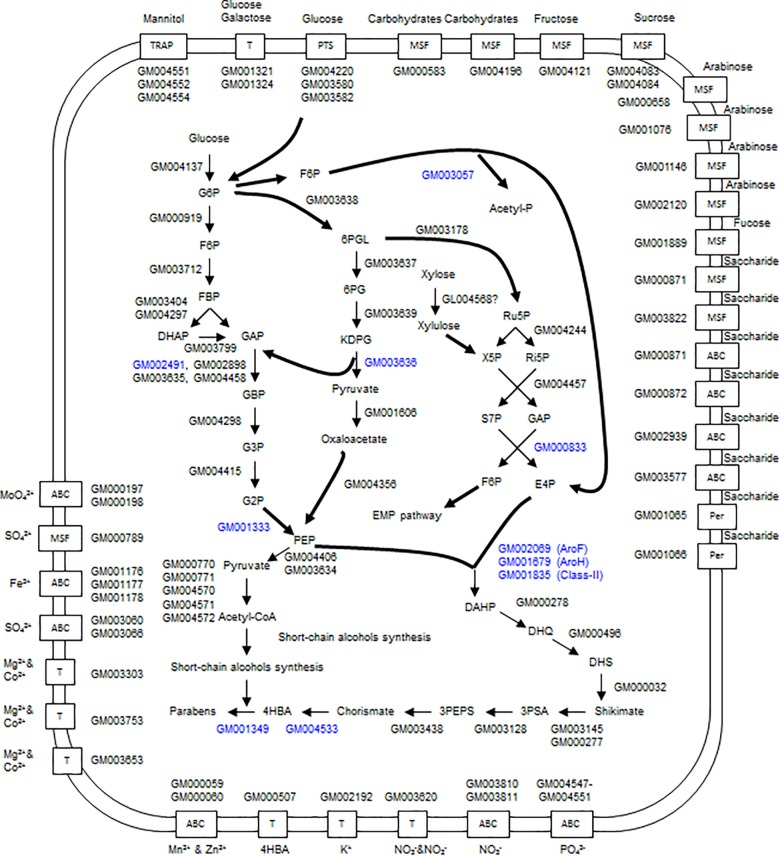
Overview of the glucose catabolism pathways and transport systems of strain A4B-17. 4HBA and parabens are produced through the shikimate pathway. G6P, glucose-6-phosphate; F6P, fructose-6-phosphate; FBP, fructose-1,6-bisphosphate; DHAP, deoxy-arabinoheptulosonate-7-phosphate; GAP, glyceraldehyde-3-phosphate; GBP, glycerate-1,3-diphosphate; G3P, glycerate-3-phosphate; G2P, glycerate-2-phosphate; PEP, phosphoenolpyruvate; 6PGL, D-glucono-1,5-lactone 6-phosphate; 6PG, 6-phospho-D-gluconate; KDPG, 2-dehydro-3-deoxy-6-phospho-D-gluconate; Ru5P, ribose-5-phosphate; X5P, xylulose-5-phosphate; Ri5P, ribose-5-phosphate; S7P, sedoheptulose-7-phosphate; E4P, erythrose-4-phosphate; DHQ, 3-dehydroquinate; DHS, 3-dehydroshikimate; 3PSA, 3-phosphoshikimate; 3PEPS, 3-phospho-5-enoylpyruvylshikimate; and 4HBA, 4-hydroxybenzoate.

There are two systems for glucose import in the strain A4B-17 genome; one is the phosphoenolpyruvate (PEP)-dependent phosphotransferase system (PTS). GM004220 encodes PEP-utilizing enzyme I (PtsP); GM003582 encodes HPr protein; and GM003580 encodes enzyme II. Another system is the two permeases encoded by GM001324 and GM001321, which are 53.3% similar. The genes coding for the EMP and HMP pathways exist in the genome, but they do not constitute a cluster because they are scattered throughout the genome (Figure 5 and Supplementary Table S1).

**FIGURE 6:**
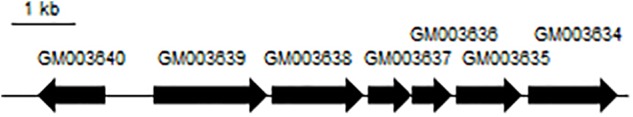
The ED pathway operon of strain A4B-17. Enzymes: GM003640, transcription regulator; GM003639, phosphogluconate dehydratase; GM003638, glucose-6-phosphate 1-dehydrogenase; GM003637, 6-phosphogluconolactonase; GM003636, KDPG aldolase; GM003635, glyceraldehyde 3-phosphate dehydrogenase; and GM003634, a putative pyruvate kinase.

It was found that A4B-17 has all of the ED pathway genes, which constitute an operon (GM003634-003640) in the genome (Figure 6). GM003635 to GM003639 are structural genes that are responsible for the conversion of glucose-6-phosphate to pyruvate and glyceraldehyde-1,3-bisphosphate. GM003640, in the opposite direction of the structural genes, is similar to a *ccpA* regulator (Puri-Taneja et al., 2006). CcpA belongs to the GalR-LacI family of bacterial transcription regulatory proteins and functions as a DNA-binding protein, either activating or repressing a number of genes in the presence of a preferred carbon source (Loll et al., 2007). CcpA uses the Ser^46^-phosphorylated form of the histidine-containing protein (HPr) as a co-regulator. Upon binding HPr, CcpA becomes activated and can bind to the *cre* site, which is near the transcription start site (Loll et al., 2007). In the GM003582 (HPr) amino acid sequence, the Ser^46^ is conserved, indicating that GM003582 may function as a co-regulator for the ED pathway operon. In *E. coli* (Peekhaus and Conway, 1998), *Zymomonas mobilis* ZM4 (Seo et al., 2005), and *P. putida* KT2440 (Chavarría et al., 2013), the ED pathway genes do not constitute an operon. Strain A4B-17 is a rare case that has a complete ED pathway operon, suggesting that a tight regulation system exists in its glucose metabolism.

Two transporters, GM001324 and GM001321, have 48.9 and 60.3% identity to GluP of *Brucella abortus* strain 2308. GluP as well as GM001324 and GM001321 exhibit the 12 transmembrane segments that are typical of members of the major facilitator superfamily (MFS) (Essenberg et al., 1997). GluP is responsible for glucose and galactose uptake in *B. abortus*. GM004554, GM004552, and GM004551 were assigned to a tripartite ATP-independent periplasmic (TRAP)-type mannitol/chloroaromatic compound transport system in which they were the periplasmic, large permease and small permease components, respectively. The TRAP transporters use the energy of an electrochemical ion gradient to drive uphill substrate transport (Pernil et al., 2010; Mulligan et al., 2011). The substrates of the characterized TRAP transporters are monocarboxylate and aromatic compounds and sugars. There are many permeases belonging to the major facilitator superfamily. They are similar to the permeases that are involved in carbohydrate transport.

The shikimate pathway is a seven-step metabolic route used by bacteria, fungi, algae, parasites, and plants for the biosynthesis of chorismate, which is an important intermediate in the biosynthesis of aromatic amino acids, indole, 2,3-dihydroxybenzoic acid, salicylic acid, alkaloids, 4-aminobenzoate, vitamin K, folate, ubiquinone, and various secondary metabolites. The condensation of PEP from the glycolysis pathway and ethyrose-4-phosphate (E4P) from the pentose phosphate pathway is the first step, catalyzed by 3-deoxy-D-arabino-heptulosonate 7-phosphate (DAHP) synthase. Until now, two distinct classes of DAHP synthase have been described, one restricted to enzymes from microorganisms (class-I) and the other exclusively composed of enzymes from plants (class-II). Many members of bacteria possess two classes; class-II DAHP synthase have been shown to be specifically dedicated to secondary metabolite synthesis (Chen et al., 1999; Silakowski et al., 2000; He et al., 2006; Light and Anderson, 2013). Chorismate was the prime allosteric effector, and tryptophan was found to be a minor feedback inhibitor for class-II DAHP synthase (Gosset et al., 2001). *E. coli* has three class-I DAHP synthase isozymes, AroF, AroG, and AroH, which are sensitive to tyrosine, phenylalanine, and tryptophan, respectively. Carbon flow through the shikimate pathway is initially regulated through the repression and feedback inhibition by these three amino acids, and the latter is quantitatively the major control mechanism *in vivo* (Kikuchi et al., 1997). All shikimate pathway genes are found in the genome of strain A4B-17 but are distributed throughout the genome and thus do not constitute an operon. Strain A4B-17 has three DAHP synthase genes; two of them, GM002069 and GM001679, have 57.7 and 47.8% identity with *E. coli aroF* and *aroH*, respectively, indicating that these two DAHP synthases belong to class-I. The other DAHP synthase is GM001835, which is thought to be a class-II DAHP synthase because it has 51.1 and 49.0% identity with the DAHP synthases of *A. thaliana* (Keith et al., 1991) and tobacco (Wang et al., 1991), respectively. No gene was found that was related to *E. coli aroG*.

A 4-hydroxycinnamoyl-CoA lyase (HCHL) gene is not found in the genome sequence, indicating there is no HCHL route to 4HBA synthesis in strain A4B-17. Chorismate lyase is the enzyme that transforms chorismate into 4HBA and pyruvate. This enzyme catalyzes the first step in ubiquinone biosynthesis in *E. coli* and other Gram-negative bacteria. In the genome of strain A4B-17, a chorismate lyase gene (GM004533) was found that has 33.0% identity with *E. coli ubiC* (Nichols and Green, 1992). UbiC is not only for ubiquinone biosynthesis but also for that of 4HBA derivatives (Stadthagen et al., 2005). It is thought that GM004533 plays an important role in 4HBA biosynthesis in strain A4B-17.

Our experiments indicated that parabens are produced by the esterification of 4HBA with the appropriate alcohols, such as methyl, ethyl, propyl, butyl, heptyl, and nonyl alcohols, and these reactions may be catalyzed by ester synthases/carboxylesterases. Until now, there have been no reports related to these genes and enzymes. The ability of various species of *Enterobacter*, *Alcaligenes*, *Pseudomonas*, *Cladosporium*, *Burkholderia* (Amin et al., 2010), and *Aspergillus* (Koseki et al., 2010) to utilize parabens for growth has been reported. *Enterobacter cloacae* EM was isolated from a contaminated batch of a commercial mineral supplement that is normally well stabilized with a mixture of methyl and propyl parabens. A gene, *prbA*, which codes for the carboxylesterase (PrbA), contributed resistance to strain EM by hydrolyzing parabens. Methyl, ethyl, propyl, and butyl parabens were hydrolyzed by purified PrbA, and the specific activity was greatest with ethyl paraben (Valkova et al., 2002; Valkova et al., 2003). On the other hand, the product of a related gene, *pnbA*, which encodes a *p*-nitrobenzyl esterase (PnbA), catalyzes the hydrolysis of several β-lactam antibiotic *p*-nitrobenzyl esters to their corresponding free acids and *p*-nitrobenzyl alcohol; this protein was isolated from *B. subtilis* (Zock et al., 1994).

GM001349 has 28.8 and 32.8% identity with PrbA and PnbA at the amino acid sequence level, respectively, but the strain A4B-17 does not hydrolyze parabens; it is proposed that GM001349 is an ester synthase/carboxylesterase that is responsible for the parabens’ synthesis. Its exact functions remain to be elucidated. Based on substrate utilization data and supported by primary sequence identity, four subclasses or carboxyesterases have been characterized and termed type-A, B, C, and D (Crepin et al., 2004). GM001349, PrbA, and PnbA are analogous to other type-B carboxyesterases, which are mainly of eukaryotic origin.

Paraben biosynthesis needs short-chain alcohols for the esterification. Methanol is one of the most important platform chemicals produced by the chemical industry. Methanol could be produced as a by-product in alcohol fermentation; however, there are no reports of methanol biosynthesis from glucose by microorganisms. Two methanol biosynthesis routes are proposed, based on the KEGG pathway database. Pyruvate formate-lyase catalyzes the breaking of pyruvate to generate acetyl-CoA and formate; the latter is then reduced to methanol via formaldehyde. The other route is from pyruvate and via acetyl-CoA, acetoacetyl-CoA, acetoacetate, acetone, and methyl acetate. The conversion of methyl acetate to methanol is catalyzed by the hydrolase, and four genes that code for this enzyme are found on the strain A4B-17 genome sequence.

It is thought that the propanoate metabolism pathway (KEGG map00640) is responsible for the 1-propanol synthesis, and a series of genes related to this pathway are found on the genome sequence. 1-Propanol has the potential to be used as a fuel substitute and a feasible precursor of propylene, which is as monomer of the general use polymer polypropylene. No microorganisms have been identified that produce propanol from glucose in industrially relevant qualities, although small amounts have been identified as microbial by-products (Janssen, 2004). Two artificial pathways for synthesizing 1-propanol from glucose have been reported. One employs 2-ketobutyrate as an intermediate via a threonine pathway or a citramalate pathway, and the other uses 1,2-propanediol as an intermediate (Urano et al., 2015).

**FIGURE 7:**
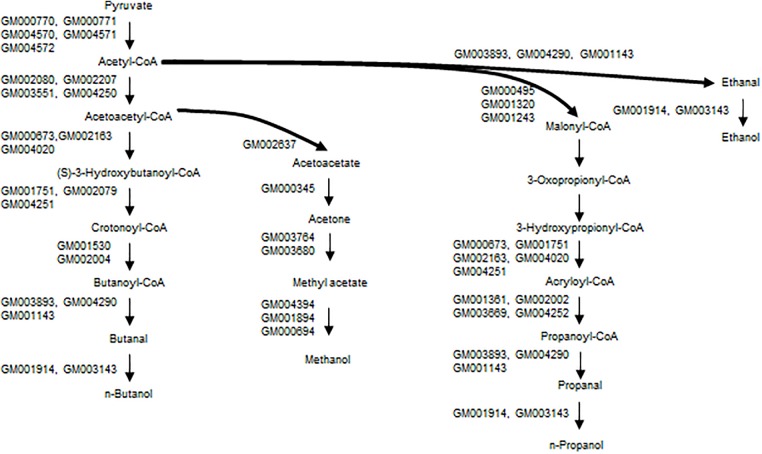
Proposed pathways for short-chain alcohols of strain A4B-17.

Ethanol could be produced via acetyl-CoA and acetaldehyde, and genes that are involved in this pathway are found on the genome sequence (Figure 7). For butanol synthesis, a typical butanol fermentation pathway (KEGG map00650) is found in the genome sequence. It is difficult to identify any pathways for n-heptyl and n-nonyl alcohol synthesis. While there are no reports of odd alcohol biosynthesis in bacteria, a mutational library analysis will be carried out in the future.

In this work, we provided the whole genome sequence of *Microbulbifer* sp. A4B-17, and annotated sugar utilization pathways. All genes responsible for the 4HBA and parabens synthesis were found on the genome. The mechanism for the paraben synthesis was elucidated by physiological and biochemical experiments. This project is fundamental research on 4HBA and paraben biosynthesis from renewable resources.

## Author Contributions

JT, ZF, and XP performed the genome analysis. LZ and WW performed the DNA manipulation. LPZ performed the RNA manipulation. ZL, QZ, and KX conducted the HPLC analysis.

## Conflict of Interest Statement

The authors declare that the research was conducted in the absence of any commercial or financial relationships that could be construed as a potential conflict of interest.

## Supplementary Material

FIGURE S1 Circular overview of the A4B-17 strain genome. From outward to inward: genes annotated by Swiss-Prot (circle 1), GO (circle 2), KEGG (circle 3), COG (circle 4), non-coding RNA (circle 5), GC content (circle 6), and GC skew (circle 7) are shown.

TABLE S1 Major genes involved in this study.
