# Supplementary figures and images for: Genomic Analysis of Microbulbifer sp. Strain A4B-17 and the Characterization of Its Metabolic Pathways for 4-Hydroxybenzoic Acid Synthesis
Source: Front Microbiol. 2018 Dec 18;9:3115. doi: 10.3389/fmicb.2018.03115 (PMC6305291; doi:10.3389/fmicb.2018.03115)

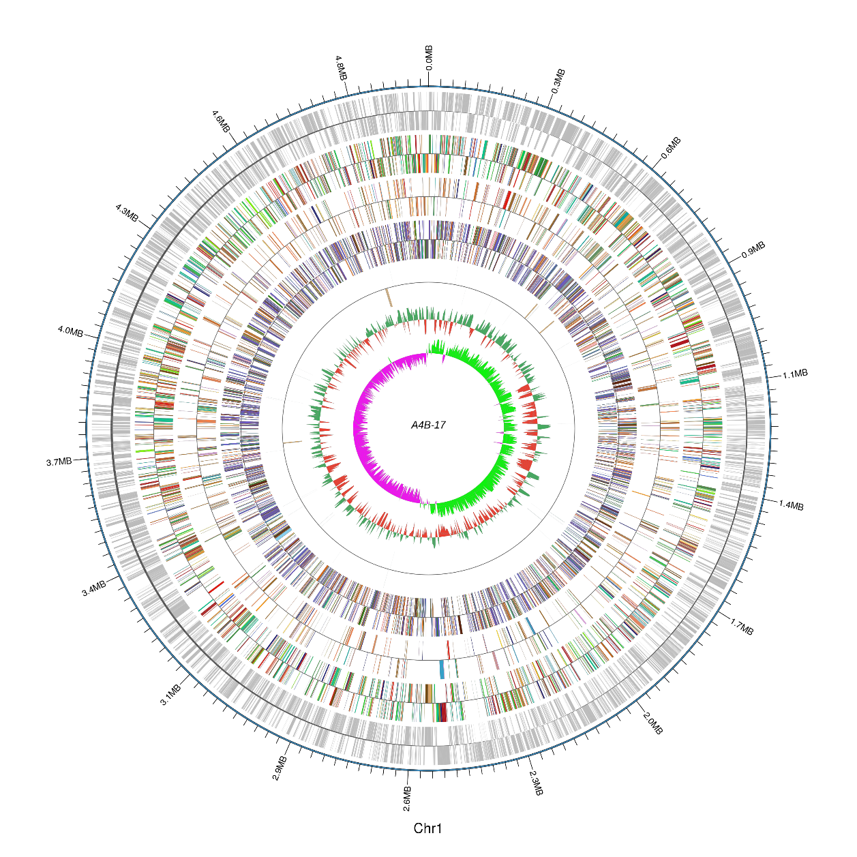

Supplement: FIGURE S1 — Circular overview of the A4B-17 strain genome. From outward to inward: genes annotated by Swiss-Prot (circle 1), GO (circle 2), KEGG (circle 3), COG (circle 4), non-coding RNA (circle 5), GC content (circle 6), and GC skew (circle 7) are shown. [file Image_1.TIF]
