# Supplementary material for: Genomic Analysis of Microbulbifer sp. Strain A4B-17 and the Characterization of Its Metabolic Pathways for 4-Hydroxybenzoic Acid Synthesis
Source: Front Microbiol. 2018 Dec 18;9:3115. doi: 10.3389/fmicb.2018.03115 (PMC6305291; doi:10.3389/fmicb.2018.03115)
Supplement: TABLE S1 — Major genes involved in this study. [file Table_1.DOCX]

Supplementary table 1. Major genes involed in this study

| **Locus tag** | **Common name** | **EC no.** | **Pathway** |
| --- | --- | --- | --- |
| GM004137 | Glucokinase | 2.7.1.2 | EMP |
| GM000919 | Glucose-6-phosphate isomerase | 5.3.1.9 | EMP |
| GM003712 | 6-Phosphofructokinase | 2.7.1.11 | EMP |
| GM003404 | Fructose-bisphosphate aldolase | 4.1.2.13 | EMP |
| GM004297 | Fructose-bisphosphate aldolase | 4.1.2.13 | EMP |
| GM003799 | Triosephosphate isomerase | 5.3.1.1 | EMP |
| GM002491 | Glyceraldehyde 3-phosphate dehydrogenase | 1.2.1.12 | EMP |
| GM002898 | Glyceraldehyde 3-phosphate dehydrogenase | 1.2.1.12 | EMP |
| GM003635 | Glyceraldehyde 3-phosphate dehydrogenase | 1.2.1.12 | EMP |
| GM004458 | Glyceraldehyde 3-phosphate dehydrogenase | 1.2.1.12 | EMP |
| GM004298 | Phosphoglycerate kinase | 2.7.2.3 | EMP |
| GM004415 | Phosphoglycerate mutase | 5.4.2.1 | EMP |
| GM001333 | Enolase | 4.2.1.11 | EMP |
| GM004406 | Pyruvate kinase | 2.7.1.40 | EMP |
| GM003634 | Pyruvate kinase | 2.7.1.40 | EMP |
| GM003178 | 6-Phosphogluconate dehydrogenase | 1.1.1.44 | HMP |
| GM004244 | Ribose 5-phosphate isomerase | 5.3.1.6 | HMP |
| GM004457 | Transketolase | 2.2.1.1 | HMP |
| GM000833 | Transaldolase | 2.2.1.2 | HMP |
| GM003638 | Glucose-6-phosphate 1-dehydrogenase | 1.1.1.49 | ED and HMP |
| GM003637 | 6-Phosphogluconolactonase | 3.1.1.31 | ED |
| GM003639 | Phosphogluconate dehydratase | 4.2.1.12 | ED |
| GM003636 | 2-Dehydro-3-deoxyphosphogluconate aldolase | 4.1.2.14 | ED |
| GM001606 | Pyruvate/oxaloacetate carboxyltransferase | 6.4.1.1 | Pyruvate metabolism |
| GM004356 | Phosphoenolpyruvate carboxykinase | 4.1.1.38 | Pyruvate metabolism |
| GM000770 | Pyruvate dehydrogenase E1 component | 2.3.1.12 | Pyruvate metabolism |
| GM000771 | Pyruvate dehydrogenase E2 component | 1.2.4.1 | Pyruvate metabolism |
| GM004570 | Pyruvate dehydrogenase E2 component | 2.3.1.12 | Pyruvate metabolism |
| GM004571 | Pyruvate dehydrogenase E1 component | 1.2.4.1 | Pyruvate metabolism |
| GM004572 | TPP-dependent pyruvate or acetoin dehydrogenase alpha subunit | 1.2.4.1 | Pyruvate metabolism |
| GM003057 | Fructose-6-phosphate phosphoketolase | 4.1.2.22 |  |
| GM002069 | 3-Deoxy-7-phosphoheptulonate synthase | 2.5.1.54 | Shikimate pathway |
| GM001679 | 3-Deoxy-7-phosphoheptulonate synthase | 2.5.1.54 | Shikimate pathway |
| GM001835 | 3-Deoxy-7-phosphoheptulonate synthase | 2.5.1.54 | Shikimate pathway |
| GM000278 | 3-Dehydroquinate synthase | 4.2.3.4 | Shikimate pathway |
| GM000496 | 3-Dehydroquinate dehydratase | 4.2.1.10 | Shikimate pathway |
| GM000032 | Shikimate dehydrogenase | 1.1.1.25 | Shikimate pathway |
| GM003145 | Shikimate kinase | 2.7.1.71 | Shikimate pathway |
| GM000277 | Shikimate kinase | 2.7.1.71 | Shikimate pathway |
| GM003128 | 3-Phosphoshikimate 1-carboxyvinyltransferase | 2.5.1.19 | Shikimate pathway |
| GM003438 | Chorismate synthase | 4.2.3.5 | Shikimate pathway |
| GM004533 | Chorismate pyruvate lyase | 4.1.3.40 | Shikimate pathway |
| GM001349 | Ester synthase/Carboxylesterase | 3.1.1.1 | Shikimate pathway |
| GM004554 | TRAP-type mannitol/chloroaromatic compound transport system, periplasmic component |  | Sugar transport |
| GM004551 | TRAP-type mannitol/chloroaromatic compound transport system, small permease component |  | Sugar transport |
| GM004552 | TRAP-type mannitol/chloroaromatic compound transport system, large permease component |  | Sugar transport |
| GM001321 | Glucose/galactose transporter |  | Sugar transport |
| GM001324 | Glucose/galactose transporter |  | Sugar transport |
| GM004220 | Phosphoenolpyruvate-protein phosphotransferase PtsP |  | Sugar transport |
| GM003582 | HPr protein |  | Sugar transport |
| GM003580 | PEP-utilizing enzyme II |  | Sugar transport |
| GM000583 | Arabinose efflux permease, MFS family |  | Sugar transport |
| GM004196 | Permease of the drug/metabolite transporter (DMT) superfamily |  | Sugar transport |
| GM004121 | Na^+^/melibiose symporter or related transporter |  | Sugar transport |
| GM004084 | ABC-type sugar transport system, ATPase component |  | Sugar transport |
| GM004083 | ABC-type sugar transport system, ATPase component |  | Sugar transport |
| GM000495 | Acetyl-CoA carboxylase/biotin carboxylase | 6.4.1.2 | [Propanoate metabolism](http://www.genome.jp/kegg-bin/show_pathway?map=map00640&show_description=show) |
| GM001320 | Acetyl-CoA carboxylase/biotin carboxylase | 6.4.1.2 | [Propanoate metabolism](http://www.genome.jp/kegg-bin/show_pathway?map=map00640&show_description=show) |
| GM001243 | Acetyl-CoA carboxylase/biotin carboxylase | 6.4.1.2 | [Propanoate metabolism](http://www.genome.jp/kegg-bin/show_pathway?map=map00640&show_description=show) |
| GM000673 | Enoyl-CoA hydratase | 4.2.1.17 | [Propanoate metabolism](http://www.genome.jp/kegg-bin/show_pathway?map=map00640&show_description=show) |
| GM001751 | Enoyl-CoA hydratase | 4.2.1.17 | [Propanoate metabolism](http://www.genome.jp/kegg-bin/show_pathway?map=map00640&show_description=show) |
| GM002163 | Enoyl-CoA hydratase | 4.2.1.17 | [Propanoate metabolism](http://www.genome.jp/kegg-bin/show_pathway?map=map00640&show_description=show) |
| GM004020 | Enoyl-CoA hydratase | 4.2.1.17 | [Propanoate metabolism](http://www.genome.jp/kegg-bin/show_pathway?map=map00640&show_description=show) |
| GM004251 | Enoyl-CoA hydratase | 4.2.1.17 | [Propanoate metabolism](http://www.genome.jp/kegg-bin/show_pathway?map=map00640&show_description=show) |
| *GM001361* | Acyl-CoA dehydrogenase | 1.3.99.3 | [Propanoate metabolism](http://www.genome.jp/kegg-bin/show_pathway?map=map00640&show_description=show) |
| *GM002002* | Acyl-CoA dehydrogenase | 1.3.99.3 | [Propanoate metabolism](http://www.genome.jp/kegg-bin/show_pathway?map=map00640&show_description=show) |
| *GM003669* | Acyl-CoA dehydrogenase | 1.3.99.3 | [Propanoate metabolism](http://www.genome.jp/kegg-bin/show_pathway?map=map00640&show_description=show) |
| *GM004252* | Acyl-CoA dehydrogenase | 1.3.99.3 | [Propanoate metabolism](http://www.genome.jp/kegg-bin/show_pathway?map=map00640&show_description=show) |
| GM002637 | CoA-transferase | 2.8.3. | [Methanol metabolism](http://www.genome.jp/kegg-bin/show_pathway?map=map00640&show_description=show) |
| GM000345 | Acetoacetate decarboxylase | 4.1.1. | [Methanol metabolism](http://www.genome.jp/kegg-bin/show_pathway?map=map00640&show_description=show) |
| GM003764 | Acetone monooxygenase | 1.14.13 | [Methanol metabolism](http://www.genome.jp/kegg-bin/show_pathway?map=map00640&show_description=show) |
| GM003680 | Acetone monooxygenase | 1.14.13 | [Methanol metabolism](http://www.genome.jp/kegg-bin/show_pathway?map=map00640&show_description=show) |
| GM004394 | Methyl acetate hydrolase | 3.1.1- | [Methanol metabolism](http://www.genome.jp/kegg-bin/show_pathway?map=map00640&show_description=show) |
| GM001894 | Methyl acetate hydrolase | 3.1.1 | [Methanol metabolism](http://www.genome.jp/kegg-bin/show_pathway?map=map00640&show_description=show) |
| GM000694 | Methyl acetate hydrolase | 3.1.1 | [Methanol metabolism](http://www.genome.jp/kegg-bin/show_pathway?map=map00640&show_description=show) |
| GM002080 | Acetyl-CoA C-acetyltransferase | 2.3.1.9 | Butanoate metabolism |
| GM002207 | Acetyl-CoA C-acetyltransferase | 2.3.1.9 | Butanoate metabolism |
| GM003551 | Acetyl-CoA C-acetyltransferase | 2.3.1.9 | Butanoate metabolism |
| GM004250 | Acetyl-CoA C-acetyltransferase | 2.3.1.9 | Butanoate metabolism |
| GM001751 | 3-Hydroxyacyl-CoA dehydrogenase | 1.1.1.35 | Butanoate metabolism |
| GM002079 | 3-Hydroxyacyl-CoA dehydrogenase | 1.1.1.35 | Butanoate metabolism |
| GM004251 | 3-Hydroxyacyl-CoA dehydrogenase | 1.1.1.35 | Butanoate metabolism |
| GM000673 | Enoyl-CoA hydratase | 4.2.1.17 | Butanoate metabolism |
| GM002163 | Enoyl-CoA hydratase | 4.2.1.17 | Butanoate metabolism |
| GM004020 | Enoyl-CoA hydratase | 4.2.1.17 | Butanoate metabolism |
| GM001530 | Butyryl-CoA dehydrogenase | 1.3.8.1 | Butanoate metabolism |
| GM002004 | Trans-2-enoyl-CoA reductase (NAD^+^) | 1.3.1.44 | Butanoate metabolism |
| GM003893 | Aldehyde dehydrogenase (NAD^+^) | 1.2.1.3 | Butanoate metabolism |
| GM004290 | Aldehyde dehydrogenase (NAD^+^) | 1.2.1.3 | Butanoate metabolism |
| GM001143 | Aldehyde dehydrogenase (NAD(P)^+^) | 1.2.1.5 | Butanoate metabolism |
| GM001914 | Oxidoreductase | 1.1.1 | Butanoate metabolism |
| GM003143 | Oxidoreductase | 1.1.1 | Butanoate metabolism |
